# *ArabidopsisKCS5* and *KCS6* Play Redundant Roles in Wax Synthesis

**DOI:** 10.3390/ijms23084450

**Published:** 2022-04-18

**Authors:** Haodong Huang, Asma Ayaz, Minglü Zheng, Xianpeng Yang, Wajid Zaman, Huayan Zhao, Shiyou Lü

**Affiliations:** 1 State Key Laboratory of Biocatalysis and Enzyme Engineering, School of Life Sciences, Hubei University, Wuhan 430062, China; haodonghuang@stu.hubu.edu.cn (H.H.); asmaayaz@bs.qau.edu.pk (A.A.); 202021107010975@stu.hubu.edu.cn (M.Z.); 2 College of Life Sciences, Shandong Normal University, Jinan 250014, China; yangxp2006@sdnu.edu.cn; 3 Department of Life Sciences, Yeungnam University, Gyeongsan 38541, Korea; shangla123@gmail.com; 4 Hubei Hongshan Laboratory, Wuhan 430070, China

**Keywords:** *KCS*, wax synthesis, drought, expression

## Abstract

*3-ketoacyl-CoA synthases (KCSs),* as components of a fatty acid elongase (FAE) complex, play key roles in determining the chain length of very-long-chain fatty acids (VLCFAs). *KCS6,* taking a predominate role during the elongation from C26 to C28, is well known to play an important role in wax synthesis. *KCS5* is one paralog of *KCS6* and its role in wax synthesis remains unknown. Wax phenotype analysis showed that in *kcs5* mutants, the total amounts of wax components derived from carbon 32 (C32) and C34 were apparently decreased in leaves, and those of C26 to C32 derivatives were obviously decreased in flowers. Heterologous yeast expression analysis showed that *KCS5* alone displayed specificity towards C24 to C28 acids, and its coordination with *CER2* and *CER26* catalyzed the elongation of acids exceeding C28, especially displaying higher activity towards C28 acids than *KCS6*. BiLC experiments identified that *KCS5* physically interacts with *CER2* and *CER26*. Wax phenotype analysis of different organs in *kcs5* and *kcs6* single or double mutants showed that *KCS6* mutation causes greater effects on the wax synthesis than *KCS5* mutation in the tested organs, and simultaneous repression of both protein activities caused additive effects, suggesting that during the wax biosynthesis process, *KCS5* and *KCS6* play redundant roles, among which *KCS6* plays a major role. In addition, simultaneous mutations of two genes nearly block drought-induced wax production, indicating that the reactions catalyzed by *KCS5* and *KCS6* play a critical role in the wax biosynthesis in response to drought.

## 1. Introduction

The evolution of structural features facilitated plants to adapt to harsh environmental conditions by exudation of waxy substances to the surface [1,2]. Waxes coating the plant surface prevent desiccation [3], UV light, and frost damage [4], as well as provide a barrier against pathogens [5] and plant–insect interactions [6]. Wax consists of very-long-chain fatty acids (VLCFAs) and their derivatives. Wax production initially starts from plastid where long-chain fatty acids (LCFAs) are generated by a fatty acid synthetase (FAS); then, the generated products are transported into the endoplasmic reticulum (ER) and elongated to very-long-chain fatty acids (VLCFAs) by a fatty acid elongase (FAE) complex. Consequently, the elongated products are further modified via an alcohol-forming pathway and aldehyde-forming pathway into primary alcohols, esters, aldehydes, alkanes, secondary alcohols, and ketones. During the wax synthesis process, VLCFAs play a critical role in the production of cuticular wax [7]. 

VLCFA synthesis is proceeded by the sequential condensation of two carbons via four consecutive enzymatic reactions in the endoplasmic reticulum (ER) [8,9], including condensation, reduction, dehydration, and second reduction. These reactions are catalyzed by a fatty acid elongase complex including *3-KETOACYL-COA SYNTHASE* (*KCS*), *PASTICCINO2* (*PAS2*), *ENOYL-COA REDUCTASE* (*ECR*), and *β-KETOACYL REDUCTASE* (*KCR1*), among which *KCSs* are key rate-limiting condensing enzymes in the fatty acid biosynthesis and determine substrate specificity [10,11]. *KCSs* usually present as multiple copies among different species [12,13,14], and in *Arabidopsis*, 21 *KCS* genes are found, among which some *KCSs* have been identified to be specifically responsible for the production of VLCFAs with a certain chain length, providing divergent substrates for sphingolipids, membrane lipids, cuticular lipids, and storage lipids. *KCS1/KCS18* catalyzes the elongation of less than C24 acids, *KCS1* is identified to be involved in the synthesis of wax and suberin [15], and *KCS18* is required for producing the seed storage triacylglycerols (TAGs) [16]. *KCS2* and *KCS20* play redundant roles in the elongation of C20 to C22 and are involved in the synthesis of suberin and cuticular wax [17]. *KCS9* catalyzes the elongation of C22 to C24, playing multiple roles in the production of suberin, wax, and membrane lipids [18]. *KCS6*, a gene also known as *CER6*, showing activity towards C22, C24, C26, and C28, plays a dominant role in the elongation from C26 to C28 acids during wax synthesis [18,19,20,21]. *KCS6* also catalyzes the elongation of C30 and C32 but requires *CER2* and *CER26* as cofactors [22,23]. Trichome waxes often contain some extra longer components exceeding C34, the biosynthesis of which requires the involvement of *KCS16*, which catalyzes the production of C36 and C38 acids [7,14].

The dysfunction of other components of the FAE complex, except for *KCS*, often causes lethal effects [24,25,26]. However, *KCSs* are often present in multiple copies in different plants, and thus, the knockout of either one or two *KCSs* does not cause severe phenotypes, indicating the redundant roles of *KCSs* in the elongation of VLCFAs. Some *KCS* genes including *KCS1*, *2*, *6*, *9*, *10*, *16,* and *20* have been identified as required for the wax components with different chain lengths [7,14]. However, it is still unknown if other *KCSs* are also involved in this process. *KCS5* is a paralogue of *KCS6*. The role of *KCS6* in wax synthesis is well defined in different plants [21], such as *Citrus sinensis*, *Hordeum vulgare*, *Solanum tuberosum*, *Solanum lycopersicum*, *Gossypium hirsutum*, *Triticum aestivum,* and *Oryza sativa* [22,27,28,29,30,31,32], whereas the role of *KCS5* in wax production is less known, though its expression pattern and catalytic activity were studied [13,33].

To investigate the role of *KCS5* and clarify the relationship between *KCS5* and *KCS6* in wax synthesis, we created *kcs5* and *kcs6* single or double mutants by the CRISPER/Cas9 method, then carefully examined the wax phenotype of *KCS5* and *KCS6* single or double mutants in different organs and compared catalytic specificity between *KCS5* and *KCS6* in yeast and tobacco expression systems. Since the involvement of *KCS6* in the elongation of C30 and C32 acids requires the aid of *CER2* and *CER26*, it intrigued us to check the catalytic activity of *KCS5* together with *CER2* or *CER26* and examine the physical interactions between *KCS5* and *CER2* using the BiLC system. Finally, we evaluated the effects of *KCS5* and *KCS6* mutation on drought-inducible wax production. Taken together, our study identifies the role of *KCS5* in wax biosynthesis of different organs, the redundancy of *KCS5* and *KCS6* in catalyzing the elongation of C24 to C32 acids, and the synergistic effects of simultaneously suppressing both genes on wax production under normal or water deficit conditions.

## 2. Results

### 2.1. KCS5 Dysfunction Caused Different Effects on Wax Profiling of Different Organs

To create the *kcs5* mutants, one 126 bp and 125 bp fragment located in the first exon was deleted in *KCS5* genomic DNA by the CRISPER-Cas9 method, generating *kcs5-1* and *kcs5-2* mutants, respectively (Figure S1). Wax was extracted from leaves, stems, and flowers of two *kcs5* mutants and then determined by gas chromatography–mass spectrometry (GC-MS).

**Figure 1:**
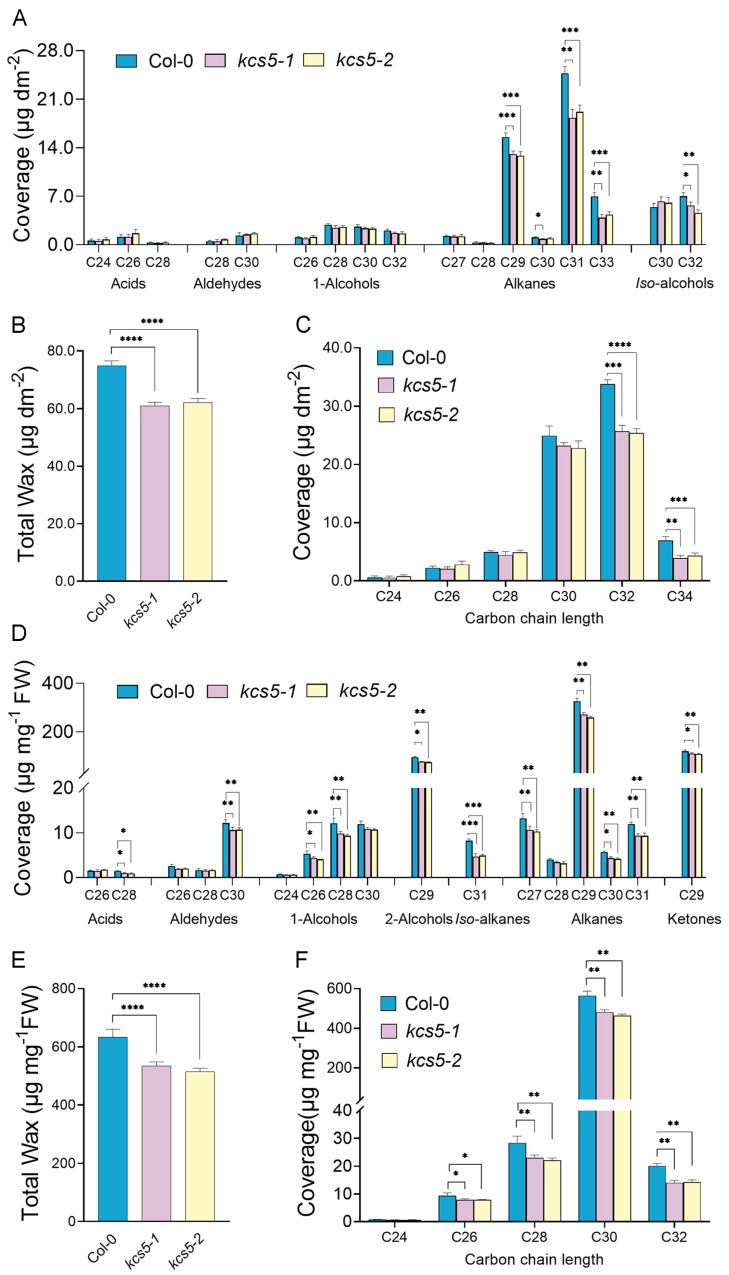
Wax profile in rosette leaves and flowers of *kcs5-1, kcs5-2* mutants, and Col-0. (**A**,**D**) Amount of each wax component in rosette leaves and flowers; wax coverage is expressed as wax amounts per stem surface area (μg.dm^−2^). Each wax constituent is designated by carbon chain length and labeled by chemical class along the x axis. (**B**,**E**) Amount of all wax components with equal carbon chain lengths in rosette leaves and flowers. (**C**,**F**) Total wax amounts. Data are means ± SE of five biological replicates. (* *p* <0.01; ** *p* < 0.05; *** *p* < 0.001, **** *p* < 0.0001).

In our study, the detectable wax components in rosette leaves consist of free fatty acids (FAs), aldehydes, primary alcohols, alkanes, and *iso*-alcohols (Figure 1A). Our experiment showed that the total wax amounts of *kcs5-1* and *kcs5-2* were significantly decreased, reduced to 81.4% and 83.0% that of wild-type plants, respectively (Figure 1B). The alteration of total wax amounts was mainly attributed to the great reduction in alkanes (Figure 1A, Table S1). Alkanes take predominate roles in leaf waxes and the total alkanes of *kcs5-1* and *kcs5-2* leaves were reduced by 24.6% and 22.4%, respectively, among which C29, C31, and C33 alkanes were significantly decreased. Apart from the straight-chain waxes, two branched-chain wax components were also detected in rosette leaves, including C30 and C32 *iso*-alcohols, among which C32 *iso*-alcohols were obviously decreased in *kcs5* mutants. To investigate if *KCS5* is specifically responsible for the elongation of some VLCFAs with certain chain lengths, we arranged the derivatives from the same VLCFA altogether. Our results showed that *KCS5* knockout caused the significant reduction in components with chain length exceeding C30 in rosette leaves, suggesting that the elongation of VLCFAs beyond C30 is impaired in *kcs5* mutant rosette leaves (Figure 1C).

Similar to rosette leaves, the flower waxes of *kcs5* mutants were also significantly decreased (Figure 1D,E). In *kcs5* mutant flowers, most of the wax components were apparently decreased, which caused a significant reduction in total wax amounts. The total wax amounts of *kcs5-1* and *kcs5-2* were decreased by 15.6% and 18.5%, respectively, which is due to the decrease in most wax components (Figure 1D, Table S1). As the predominant component, the total amounts of alkanes in *kcs5-1* and *kcs5-2* were reduced to 82.7% and 79.1% of Col-0, respectively, among which all alkanes with chain length ranging from C27 to C31 were decreased. As secondary dominant components, C29 secondary alcohol in *kcs5-1* and *kcs5-2* was decreased to 82.0% and 79.1%, respectively. C26 and C30 aldehydes, C26 to C30 primary alcohols, and C28 fatty acids were also decreased at different levels. Apart from these straight-chain waxes, C31 branched alkanes of *kcs5-1* and *kcs5-2* were greatly decreased to 57.0% and 59.9% of Col-0. After analyzing the amounts of derivatives with different chain lengths, we found that *KCS5* knockout caused a significant reduction in all wax components beyond C24 in flowers (Figure 1F), suggesting that the elongation of VLCFAs exceeding C24 is affected in *kcs5* mutant flowers. We also checked the stem wax profile and found that the amounts of all wax constituents in *kcs5* mutant stems were similar to that of Col-0 and consequently, the total wax amount was barely changed at all, suggesting that different from rosette leaves and flowers, the *KCS5* mutation has no visible effects on stem wax synthesis (Table S1, Figure S2).

### 2.2. KCS5 Coordinating with CER2 and CER26 Displays Broader Substrate Preferences 

**Figure 2:**
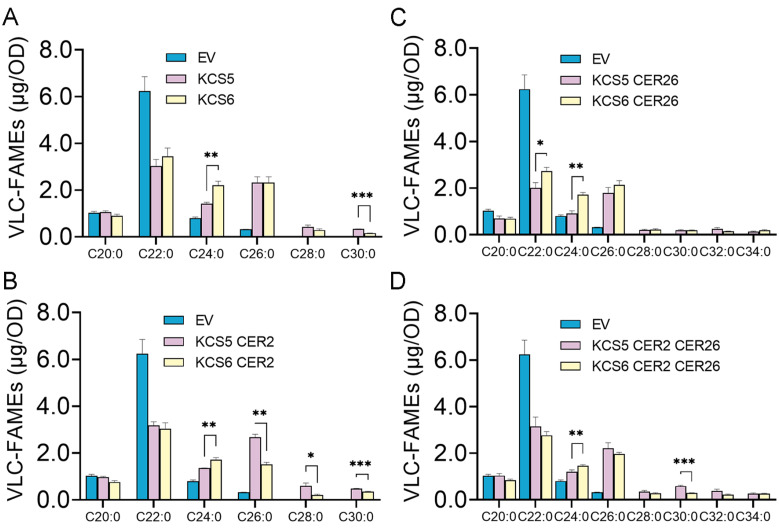
The catalytic activity of *KCS5* and *KCS6* examined by the yeast system. *KCS5*, *KCS6,* or empty vector (EV) were alone expressed or co-expressed with *CER2* and *CER26* in yeast strain *BY4741 Δelo3Δfah1,* and the generated products are displayed in the form of VLCFA-FAMEs (**A**–**D**). FAMEs were synthesized by transmethylation before GC analysis. The values shown are mean ± SD (*n* = 4). * *p* < 0.05; ** *p* < 0.01; *** *p* < 0.001.

*KCSs* often display catalytic specificity towards substrates with certain lengths. To investigate the catalytic preferences of *KCS5*, we first constructed the Δ*fah1*Δ*elo3* double mutant in which hydroxylated C22 acids (α-OH-C22:0) and VLCFAs exceeding C26 were not produced, owing to the deficiency of *fah1* and *elo3*, respectively. In the previous studies, Δ*elo3* single mutant was always used, but we found that the peak generated by α-OH-C22:0 is high and also overlaps with that of C26 acids (Figure S3). To eliminate the effects of signals generated by α-OH-C22:0, we made the double mutant Δ*fah1*Δ*elo3* and then transformed *KCS5* into yeast strain *BY4741* Δ*fah1*Δ*elo3.* Yeast expression analysis revealed that *KCS5* expression alone generated C24 to C30 acids (Figure 2). However, the levels of the generated C24 and C26 acids were apparently higher than other acids, suggesting that *KCS5* preferentially catalyzes C22 and C24, although it has relatively broad substrate preferences. *KCS6* also displays a similar catalytic property (Figure 2). 

Previous studies identified that the involvement of *KCS6* in the elongation of more than C28 acids requires the acids of *CER2* and *CER26*. As the paralogue of *KCS6*, *KCS5* might also coordinate with the two proteins during the catalyzing process. Thus, we also checked the catalytic activity of *KCS5* co-expressing with *CER2* or *CER26*. The expression of *KCS5* together with *CER2* also generates C24 to C30 acids, which is similar to that of *KCS5* expression alone. We also noticed that the co-expression of *KCS5* and *CER2* apparently produced more C28 and C30 acids than *KCS5* alone, revealing that *CER2* enhances *KCS5* catalytic capacity during the elongation process of C26 and C28 acids (Figure 2).

We also examined the products of yeast strains co-expressing *KCS5* and *CER26* and found that two new components, i.e., C32 and C34, were exclusively generated in the strains expressing *KCS5* and *CER26*, revealing that *CER26* could assist *KCS5* in catalyzing the production of VLCFAs exceeding C30. Finally, we checked the strains expressing *KCS5*, *CER2,* and *CER26* together and found that the amounts of C32 and C34 acids in yeast strains expressing three proteins were apparently more than the strain expressing *KCS5* and *CER26* (Figure 2, Table S2). The possible reason might be due to the increase in precursors. Altogether, these results show that *KCS5* coordinating with *CER2* and *CER26* displays broader substrate preferences, and *KCS5* displays a similar catalytic property as *KCS6*. However, we noticed that the strains expressing *KCS5* generate more 30 acids than the ones expressing *KCS6*, which was also identified in tobacco cells (Figure S4), while the strains expressing *KCS6* produce 24 more acids than the ones expressing *KCS5*, suggesting that *KCS5* and *KCS6* might have somewhat different substrate preferences. 

### 2.3. KCS5 Physically Interacts with CER2 and CER26

**Figure 3:**
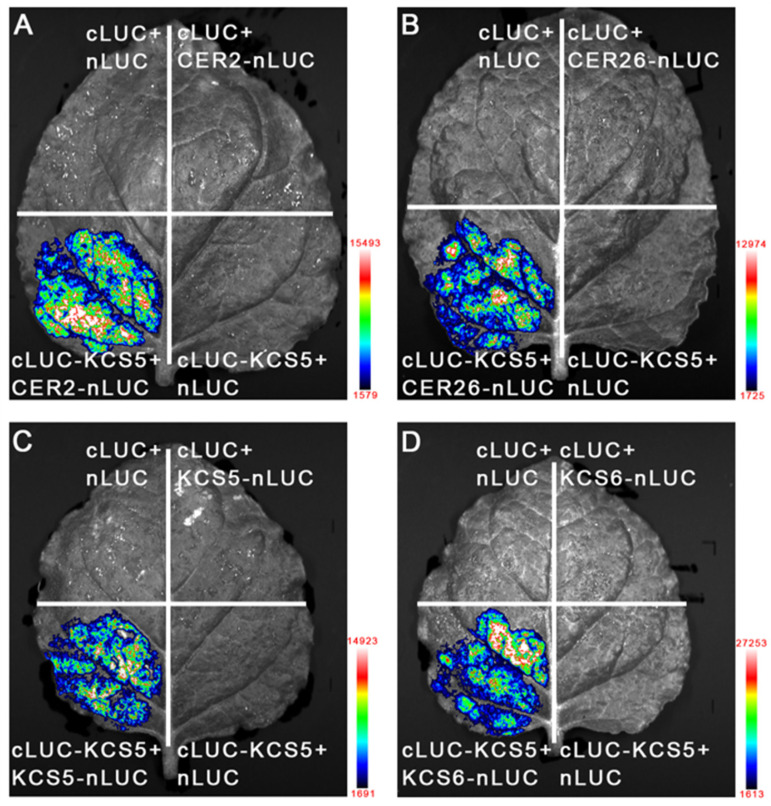
Interactions between *KCS5* and *CER2* or *CER26* by BiLC experiments. *Agrobacterium* strain *GV3101* containing different combinations (**A**–**D**) was transiently infiltrated into tobacco leaves. After 2–3 days, luminescence signals were captured by Tanon 5200 luminescence imaging system (Tianneng Technology Co., Ltd., Shanghai, China). The colors labeled beside the figures indicate signal intensity.

Previous studies showed that *KCS6* interacting with *CER2* or *CER26* in the elongation of more than C26 acids requires *CER2* and *CER2-LIKE* proteins as cofactors [34]. Our study also demonstrated that *KCS5* co-expressing with *CER2* and *CER26* resulted in the elongation of C28–C34 acids. Thus, we supposed that *KCS5* might also physically interact with *CER2* or *CER26* during its catalyzing process. To verify this possibility, we performed a bimolecular luminescence complementation (BiLC) assay using a transient transformation system. We first constructed *n-LUC* or *c-LUC* fused with *KCS5*, *KCS6*, *CER2,* and *CER26*, and then various combinations were transiently transformed into tobacco leaves. In this experiment, four combinations were used as negative controls: *cLUC* and *nLUC*, *KCS5*-cLUC and *nLUC*, *cLUC* and *nLUC-CER2,* and *cLUC* and *nLUC-CER26*. These negative controls did not display any signals, whereas two combinations—*KCS5-cLUC* and nLUC-*CER2,* and *KCS5-cLUC* and nLUC-*CER26*—showed strong LUC signals (Figure 3A,B), implicating that *KCS5* indeed interacts with *CER2* or *CER26*. Previous studies showed that *KCS* members often form homo- or heterocomplexes [35]. We also detected the interactions between *KCS5* and *KCS5*, and *KCS5* and *KCS6*, and both combinations also displayed strong signals (Figure 3C,D), indicating that they interact with each other.

### 2.4. The KCS5 and KCS6 Mutations Act Synergistically to Generate Severe Wax Defects

Both *KCS5* and *KCS6* play roles in the wax synthesis by catalyzing the elongation of C22 to C32 acids, implying that they might function redundantly during the wax production process. Their transcripts are differentially accumulated in different organs, implying that they play different roles in wax production. To evaluate the role of *KCS5* and *KCS6* and investigate the relationship between *KCS5* and *KCS6*, one G was inserted into 1788 bp of ATG downstream of *KCS6* by the CRISPER-Cas9 method in *kcs5-1* background (Figure S1), resulting in shift mutation of the open reading frame. The created mutant was named *kcs5-1 kcs6-1,* which was identified by PCR (Figure S1). To obtain *kcs6* single mutant, we also backcrossed the double mutant with Col-0, and plants only carrying the mutation site in *KCS6* were kept. We compared the phenotype of *kcs6-1* with other *KCS6* mutants and found that its phenotype resembles other identified *kcs6* mutants (previously named *cer6-1* and *cer6-2*) [20], revealing that this mutant could be used for further study. 

**Figure 4:**
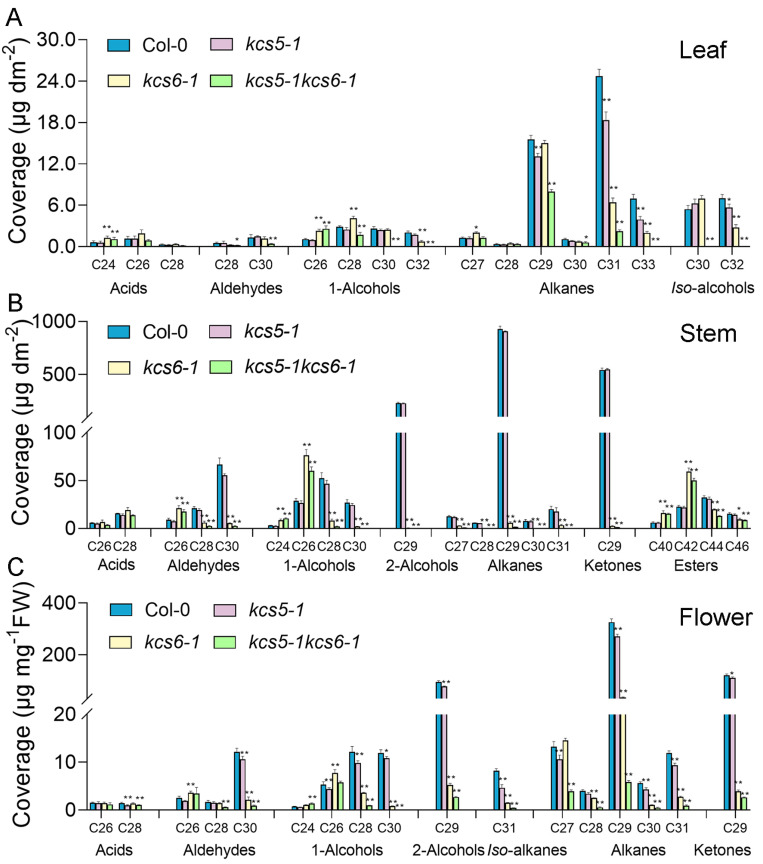
Wax profile in different organs of different plants. Rosette leaves (**A**), stems (**B**), and flowers (**C**) of *Col-0*, *kcs5-1*, *kcs6-1*, and *kcs5-1 kcs6-1* were collected for wax analysis. Wax coverage is expressed as wax amounts per leaf surface area (μg.dm^−2^). Each wax constituent was designated by carbon chain length and was labeled by chemical class along the x-axis. The values shown are means ± SD (*n* = 4). * *p* < 0.05; ** *p* < 0.01.

We carefully checked the wax phenotype in leaves, stems, and flowers of *kcs5-1 kcs6-1,* and *kcs5-1 kcs6-1* (Figure 4, Table S1). In leaves, most of the wax components exceeding C24 were moderately decreased in *kcs5-1*, which caused the slight reduction in total wax amounts in this mutant, which was decreased to 81.37% that of the wild type (Figure 4, Table S1). In *kcs6-1*, wax components exceeding C28 were significantly decreased, whereas C24 to C28 components were apparently increased (Figure 4, Table S1), revealing that C28 elongation was blocked in *kcs6-1* leaves. Since the decreasing components take dominant role in waxes, their reduction greatly decreased the total wax amounts of *kcs6-1* leaves, which were only 68.36% that of wild-type plants (Figure 4, Table S1). In *kcs5-1 kcs6-1* mutant, C24 fatty acids and C26 primary alcohols were apparently increased, though they only take minor roles in waxes, whereas most of the wax components beyond C26 including fatty acids, aldehydes, primary alcohols, and alkanes were significantly decreased, implying that C26 elongation was slightly blocked in this double mutant. In addition, we noticed that C30 and C32 primary alcohols, C33 alkanes, and *iso*-alcohols were deficient in the double mutant (Figure 4, Table S1). Since most of the wax components were significantly decreased in these mutants, the total leaf wax amounts of *kcs5-1 kcs6-1* were dramatically decreased to 25.94% that of wild-type plants. Apparently, the decline rate of *kcs6-1* is greater than that of *kcs5-1*; moreover, the decline rate of double mutant is apparently more than either single mutant, suggesting that compared with *KCS5*, *KCS6* plays a more important role in wax synthesis, and simultaneous suppression of *KCS5* and *KCS6* activity causes additive effects.

In stems, the total wax amounts of *kcs5-1* are barely changed, but those of *kcs6-1* and *kcs5-1 kcs6-1* were drastically decreased to 13.36% and 9.95% that of wild-type plants, respectively (Figure 4, Table S1). In *kcs6-1*, most wax components derived from FAs beyond C26 were greatly decreased, whereas most of the components derived from C24 and C26 FAs were significantly increased, including C26 aldehyde, C24, C26 primary alcohols, and C40 and C42 esters (Figure 4, Table S1), suggesting that the elongation of C26 acids was obviously blocked in the stem. The variation tendency of the double mutant is similar to *ksc6-1*, and the elongation of C26 acids was also blocked in the double mutant. However, we noticed that the decline rate of the double mutant is still more than *kcs6-1*, indicating that *KCS5* mutation exaggerates the mutant phenotype, though it only plays a minor role during stem wax production.

In flowers, the total wax amounts of *kcs5-1* are also moderately decreased, whereas the total wax amounts of *kcs6-1* and double mutant were sharply decreased to 14.28% and 5.23% that of wild-type plants, respectively (Figure 4, Table S1). In *kcs6-1* and double mutant, most C24 and C26 wax components including aldehydes and primary alcohols were obviously increased, whereas components exceeding C26 were apparently decreased (Figure 4, Table S1), suggesting that the elongation of C26 acids was also blocked in the flowers of *kcs6-1* and double mutant, resembling stems. The wax phenotype of the double mutant is also severer than either of the single mutants, revealing that *KCS5* and *KCS6* play redundant roles in wax synthesis.

### 2.5. Double Mutant Is Susceptible to Lose Water 

**Figure 5:**
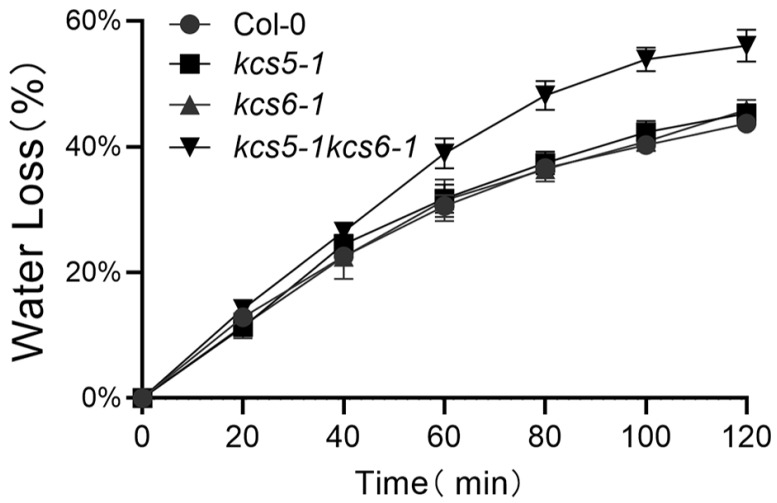
Water loss assay of detached leaves. Excised leaves’ water loss rates were recorded over 120 min and measured as a percentage of the initial weight of fully hydrated leaves. Values are the mean of five replicate assays. Error bar = SD. The experiments were repeated once with similar results.

Wax, an important component of plant cuticle, plays a vital role against water loss. Our study showed that the total leaf wax amounts of *kcs5-1*, *kcs6-1*, and *kcs5-1 kcs6-1* were decreased to 60%, 50%, and 20% that of wild-type plants, respectively (Figure 4, Table S1), implying that these mutants might have different water retention ability. We checked the water loss of detached leaves of these mutants to evaluate the water retention capacity of these plants. The water loss rates of *kcs5-1* and *kcs6-1* were similar to the wild type, but that of the double mutant was apparently higher than wild-type plants (Figure 5), revealing that maintaining certain amounts of waxes is vital for water preservation.

### 2.6. Simultaneous Mutation of KCS5 and KCS6 Nearly Blocked Drought-Induced Wax Production

**Figure 6:**
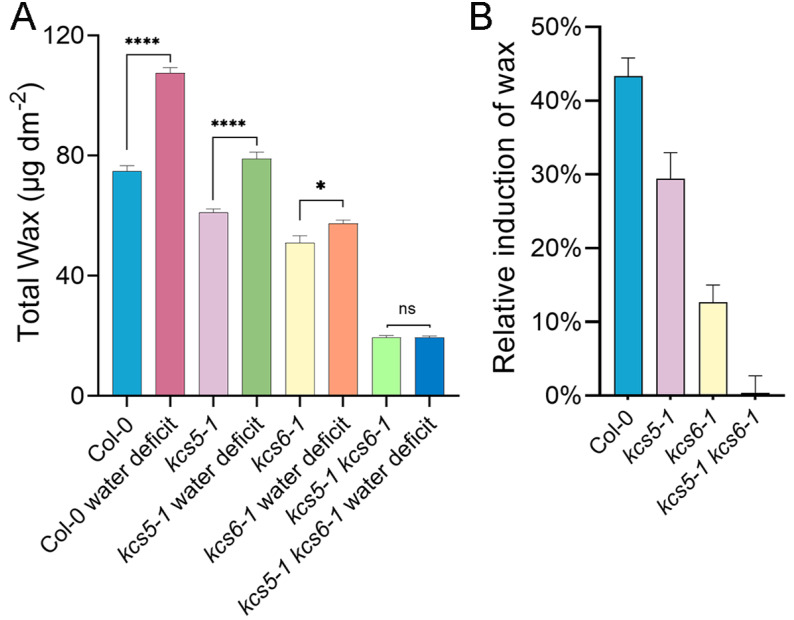
Wax production of different plants in response to water deficit conditions. Wax analysis was performed in different plants grown under normal and water deficit conditions (**A**). Total wax amounts of plants grown under different conditions are shown (**B**). The values shown are means ± SD (*n* = 4). The experiments were repeated once with similar results. * *p* < 0.05; **** *p* < 0.0001.

Drought is well known to be an important environmental cue for inducing wax production. qRT-PCR results showed that the expression of either *KCS5* or *KCS6* is dramatically induced by drought stress (Figure S5). Thus, we wanted to know if suppression of *KCS5* and *KCS6* alone or simultaneously has any effects on wax production in response to drought treatments. Under drought treatment, in Col-0, among the straight-chain wax components, only alkanes were drastically increased, including C29, C31, and C33, whereas other component amounts are barely changed. In addition, branched components including C30 and C32 *iso*-alcohols were also apparently increased. Owing to the increase in alkanes and branched components, the total wax amounts of Col-0 under drought treatment were increased by 43.3% compared with normal conditions (Figure 6 and Figure S6). In *kcs5-1* mutant, the total wax amounts under drought treatment were also increased by 29.4%, which was slightly less than that of wild-type plants (Figure 6 and Figure S6). Moreover, the increasing components in *kcs5-1* were similar to Col-0. In *kcs6-1* mutant, the total wax amounts were also induced upon drought, but the increase rate was low, only 111.2% (Figure 6 and Figure S6). In this mutant, only the amounts of C29, C31, and C33 alkanes were moderately increased, the branched component amounts were barely altered, and C28 primary alcohol was even decreased, which caused the low increase rate (Figure S6). In *kcs5-1 kcs6-1* mutant, only C24 acids and C31 alkane were slightly increased; moreover, C26 primary alcohol was not increased but greatly decreased (Figure S6). Since these components only take a minor role and their variations display opposite tendencies under drought treatment, the total wax amounts of double mutant under drought treatment were eventually almost the same as under normal conditions (Figure S6). From these results, it could be seen that the rising rate of *KCS6* was apparently lower than that of *kcs5* under drought treatment (Figure 6), implying that compared with *KCS5*, *KCS6* plays the dominant role in drought-induced wax production. Simultaneous suppression of *KCS5* and *KCS6* activity nearly blocked the wax production in response to water deficit (Figure 6), suggesting that the reactions catalyzed by *KCS5* and *KCS6* are the key steps determining the wax production of the plant in response to drought treatment.

## 3. Discussion

### 3.1. KCS5 Functions in Wax Synthesis, though It Plays Minor Role

*KCSs* catalyze the elongation of VLCFAs, providing substrates for sphingolipids, surface lipids, membrane lipids, and storage lipids, which display various expression patterns. Across these *KCSs,* some members are identified to be involved in wax synthesis, including *KCS6, KCS2, KCS20,* and *KCS9*, which are highly expressed in the epidermis [17,18,21], where many wax-related genes are abundantly accumulated [36]. *KCS5* was predicted to be involved in wax synthesis long ago [7,20], and there are three possible reasons: (1) *KCS5* coding protein sequence shares high identity with *KCS6* [20]; (2) it is specifically expressed in the epidermis (http://efp.ucr.edu, accessed on 7 October 2021); and (3) its coding protein is localized in ER [13], where the wax components were produced. However, it is still unknown how it functions in wax synthesis and in which organs it works. In this study, we identified that *KCS5* mutation causes obvious effects on the wax amounts of rosette leaves and flowers due to the decrease in alkanes, which are predominate components (Figure 1, Table S1), suggesting that *KCS5* plays a role in these two organs in *Arabidopsis*. In addition, heterologous yeast expression analysis showed that *KCS5* coordinating with *CER2* and *CER26* displayed specificity towards C24 to C32 acids (Figure 2), which are precursors for cuticular wax, sphingolipids, and storage lipids. These results provide substantial evidence that *KCS5* plays a role in wax synthesis.

Although *KCS5* plays a role in the wax synthesis, the wax phenotype of *kcs5* mutant is weak compared to *kcs6* mutants (Figure 4). Previous studies showed that *kcs6/cer6* mutants displayed glossy stems and male sterility [20,37]. However, the phenotype of *kcs5* mutants could not be observed by the naked eye, and the effects of its mutation on wax synthesis are only detected by equipment. Our wax phenotype analysis provides clear evidence identifying that *KCS5* only plays a minor role in wax synthesis compared to *KCS6*. The total wax amounts of *kcs5* mutant rosette leaves and flowers are only decreased by 18% and 16%, respectively (Figure 4, Table S1), whereas wax amounts of *kcs6* mutant leaves and flowers are decreased by 32% and 86%, respectively (Figure 4, Table S1). *KCS5* mutation has little effect on stem wax synthesis, whereas suppression of *KCS6* activity caused a significant reduction in stem wax amounts, which sharply decreased to 13% that of the wild type (Figure 4, Table S1). Taken together, *KCS5* mutation only takes minor a role, whereas *KCS6* plays a major role in wax biosynthesis. Since *KCS5* and *KCS6* almost display similar activity towards C22 to C32 acids (Figure 2), the different effects on wax amounts caused by the mutation of each gene might be closely related with the different expression levels of two genes. Our real-time PCR results revealed that *KCS5* transcripts are lowly accumulated in all tested organs except for silique (Figure S5); by contrast, *KCS6* transcripts are abundantly accumulated in all organs except for root, which is consistent with the previous results [13]. This phenomenon is also seen in other paralogue genes [38], i.e., one showing high expression levels that always plays a dominant role and the other which displays low expression levels playing a minor role.

### 3.2. KCS5 Plays More Important Role in Generation of C30 Acids than KCS6 in Rosette Leaves 

Although *KCS6* is identified to play a major role in wax synthesis, its mutation causes different effects on the wax synthesis of different organs. As compared with wild-type plants, the wax amounts were decreased by 31.64%, 86.64%, and 85.72% in leaves, stems, and flowers, respectively (Table S1). It is apparent that the decreasing rate of leaves is lower than that of other organs in *kcs6-1* mutant (Table S1). We carefully checked the leaf wax profiles and found that C29 alkanes, a predominant component, was only slightly altered in *kcs6-1* mutant; however, this component was significantly decreased in *kcs6-1* stems and flowers (Figure 4). The amounts of other components derived from C30 acids including C30 aldehyde, C30 primary alcohol, and *iso*-C30-alcohol were also not changed (Figure 4). These results suggest that *KCS6* plays a minor role in the generation of C30 acids in rosette leaves. As compared with *KCS6, KCS5* seems to play a more important role in this process since C29 alkane is significantly decreased in *kcs5* rosette leaves. Though *KCS5* is lowly expressed in rosette leaves (Figure S5), our heterologous yeast expression analysis showed that *KCS5* displays higher activity towards C28 acids than *KCS6* (Figure 2). Thus, it is possible that in *kcs6-1* mutant leaves, the loss of *KCS6* might be partially compensated by *KCS5* activity. However, *KCS5* and *KCS6* are not the sole enzymes responsible for the generation of C30 acids because certain amounts of C29 alkane are still present in leaves of *kcs5-1 kcs6-1* double mutant, even though the synthesis of the C30 primary alcohol and *iso*-C30-alcohol was totally blocked (Figure 4). It is possible that other *KCS* members might also be involved in the production of C30 acids, or that C29 alkane derives from a separate elongation pathway catalyzed by the ELO-like family of condensing enzymes [39].

### 3.3. The Role of Waxes in Water Retention Capacity

Many studies reveal that wax is closely related to water retention capacity [40,41,42,43,44]. In our experiment, water retention capacity of *kcs5-1* and *kcs6-1* single mutants is similar to wild-type plants, though the total wax amounts in *kcs5-1* and *kcs6-1* rosette leaves were significantly decreased by 18% and 32%, respectively, whereas the double mutant (with 74% decrease in total waxes) displays an enhanced water loss rate (Figure 5). Thus, it is apparent that a moderate reduction in waxes seems to not affect the water retention capacity. In addition, the increase in total waxes does not always decrease the cuticle permeability, thus enhancing water retention capacity. Some wax mutants containing more waxes display enhanced cuticle permeability. For example, *FIDDLEHEAD (FDH)/KCS10* mutant has more waxes in rosette leaves, which display enhanced cuticle permeability [45]. Thus, the higher epidermal permeability displayed by *fdh1* might be due to the distorted cuticle integrity. Therefore, to evaluate the water retention capacity of the wax-defected plant, other factors must be considered such as wax amount and composition, cuticle structure integrity, etc.

### 3.4. KCS5 and KCS6 Exert Synergistic Effects on Drought-Induced Wax Production

Wax plays an important role against drought stress, and many wax-related genes are highly induced upon drought stress. These genes are involved in the elongation of VLCFAs, such as *KCS2*, *KCR*, *CER10*, *CER2,* etc. [40,46,47]. They specifically participate in alcohol-forming pathways, such as *CER4*; and alkane-forming pathways, including *CER1, CER3*, and *WSD1* [48,49,50]; or are related with wax transport, such as *CER5* and *WBC11*. Moreover, the overexpression or mutation of these genes enhances or impairs plant resistance against drought stresses, suggesting that waxes play important roles against drought stress. *KCS5* and *KCS6* display similar catalytic activity, which are identified to be induced by drought, and the mutation of either gene hinders the drought-induced wax production process (Figure 6 and Figure S6). Moreover, we noticed that alkanes were greatly induced by water deficit treatment (Figure S6). In Col-0, *kcs5*, and *kcs*6*-1*, C29, C31, and C33 alkanes were all significantly increased since they take predominate roles in wax mixtures and their alterations greatly affect the total wax amounts (Figure S6). This finding is consistent with the previous study [46]. However, the previous study identified that besides alkanes, some other wax components including 24 and 26 acids, 26 aldehydes, 28 primary alcohol, and 29 ketone were also significantly increased [46], which is not present in our study. This difference might be related to different growth conditions, analytical instruments, and treatment methods. In double mutant, only 31 alkanes were obviously increased, which only takes a minor role, and though C24 acids were also increased (Figure S6), the significant reduction in C26 primary alcohol nearly eliminates the increase in C31 alkane and C24 acid. Thus, co-suppression of both *KCS5* and *KCS6* activity nearly block the wax synthesis in response to water deficit (Figure 6). *KCS5* and *KCS6* are both involved in drought-induction wax production, and the coordinating behaviors of both proteins play a critical role in controlling the wax biosynthesis in response to water deprivation.

## 4. Materials and Methods

### 4.1. Plant Materials and Growth Conditions

The *Arabidopsis* ecotype used in this study is Columbia-0 (Col-0). Seeds were sown in pots and grown in a greenhouse at 21–23 °C with 16 h light/8 h dark cycles. For water deficit treatment, seeds were sown in pots and watered normally for two weeks. Then, the plants were not watered for another two weeks. Rosette leaves of these plants under water deficit treatment together with those under normal conditions were used for RNA extraction or wax component analysis.

### 4.2. Construction of Yeast Mutant Strain and Plasmid 

To investigate the catalytic activity of *KCS5* or *KCS6*, the strain BY4741 *Fah1Δ ELO3Δ* was constructed in this study. The yeast strain BY4741 *Fah1Δ* was constructed as previously described [51,52]. The KanMX4 cassette containing two loxP sequences in strain BY4741 *Fah1Δ* was first deleted by expressing a CRE recombinase from the p416-Cre plasmid. The strain was then cultured in YPD for several generations to remove the p416-Cre plasmid. To delete *ELO3* in BY4741 *Fah1*Δ background, the KanMX4 cassette containing *ELO3* homologous sequence was amplified by using pUG6 plasmid as template and then transformed into BY4741 *Fah1*Δ yeast cells, generating the BY4741 *Fah1*Δ *elo3*Δ strain. Finally, the KanMX4 cassette in BY4741 *Fah1Δ elo3Δ* was removed again as described above. To construct expression vectors in yeast, using 2xSeamless Cloning Mix kit (Biomed, Beijing, China), *KCS5* or *KCS6* was cloned into p4X5 vector, and *CER2* and *CER26* were cloned into p4X4 and p4X4, respectively. Then, different combinations were transformed into the yeast BY4741 *Fah1Δ elo3Δ* mutant strain.

To make constructs used for BiLC experiments, the full lengths of *KCS5* and *KCS6* coding sequences (CDS) were cloned into pCAMBIA1300-nLUC vector through 2xSeamless Cloning Mix kit (Biomed, China), resulting in *nLUC:KCS5* and *nLUC:KCS6* constructs. Full lengths of *CER2* and *CER26* CDS were cloned into pCAMBIA1300-cLUC, generating *CER2:cLUC* and *CER26:cLUC* constructs, respectively. The primers are listed in Table S3. 

### 4.3. Obtainment of Single and Double Mutants of Arabidopsis kcs5 and kcs6 

The mutants *kcs5-1 and kcs5-2* were created by CRISPR/Cas9 technology, and the detailed procedure was previously described [53]. The mutation sites are provided in Figure S1. The Cas9 targets for *KCS5* were designed using the web CRISPR-P (http://cbi.hzau.edu.cn/cgi-bin/CRISPR2/CRISPR, accessed on 4 June 2021 [54]). Constructs carrying small guide RNA with target sequences and *Cas9* driven by a rice pUbi promoter were transformed into *Arabidopsis Col-0*. T2 generation plants with mutation in *KCS5* were further screened to remove the transgenic cassette. To obtain double mutant, we introduced a mutation site in the *KCS6* exon region by CRISPR/Cas9 technology in the *kcs5-1* mutant, and the homozygous plants containing the mutation sites in *KCS5* and *KCS6* genomic DNA sequences were kept for further study, in which the selection marker was eliminated. To obtain the *kcs6-1* mutant, the heterozygous or homozygous *kcs5-1 kcs6-1* mutants were crossed back with Col-0, and only plants containing the *KCS6* mutation site were reserved.

### 4.4. RT-PCR and Quantitative RT-PCR (RT-qPCR)

Total RNAs were extracted from different plants and different organs of Col-0 using Universal Plant Total RNA Extraction Kit (BioTeke, Beijing, China). To synthesize the first strand cDNA, 1 μg of total RNA was utilized as template using HiScript II 1st Strand cDNA Synthesis Kit (Vazyme, Nanjing, China). RT-PCR was performed with the gene-specific primers and using *ACTIN2* as an internal control. RT-qPCR was performed according to the manufacturer’s protocol (Vazyme, Nanjing, China) and *ACTIN2* was used as internal reference. The ΔC_T_ method was used to calculate the relative expression levels of each gene. The primers used in this study are listed in Table S3.

### 4.5. Cuticular Wax Analysis

Waxes were collected from the leaves, stems, and flowers of 4- and 6-week-old *Arabidopsis*. The wax composition was examined by following Lü et al. (2009) [55]. To determine wax component amounts, GC with an Agilent 8860 gas chromatograph equipped with DB-5 (30 m 0.25 mm × 0.25 μm; Agilent, Santa Clara, CA, USA) capillary column with a carrier gas helium and flame ionization detector was used. The column temperature was initially set at 80 °C and gradually increased at 40 °C min^−1^ to 200 °C, at which point the temperature remained unchanged for 10 min. The temperature was then increased gradually at 3 °C min^−1^, and finally reached 320 °C, at which point the temperature was held for 20 min. The quantification was performed based on flame ionization detector (FID) peak areas relative to the internal standard eicosanoids.

### 4.6. Yeast FAMEs Analysis

One single colony was incubated in a 5 mL appropriate SD dropout or YPD liquid medium in the shaker at 28 °C for overnight. One milliliter of overnight culture was diluted into an appropriate liquid medium till OD600 up to 0.05 and then incubated in the shaker at 28 °C for another 4 to 5 days. Ten-milliliter cultures were centrifuged at 2000 r/min for 5 min, the supernatant was discarded, and pellets were reserved. This step was repeated once. Then, the pellets were suspended in 2 mL freshly made solution containing 5% sulfuric acid in methanol (*V*/*V*), 50 μL C17:0 methyl ester (1 mg/mL), and 80 μL dimethoxypropane, and then vortexed for 30 s. Afterward, the samples were incubated in the oven at 85–90 °C for 2 h and then cooled to room temperature. The cooling samples were resuspended in 1.5 ml of 0.9% sodium chloride and 2 ml n-hexane accordingly, and vortexed vigorously for 30 s. The vortexed samples were centrifuged at 2000 r/min for 5 min, and then the upper organic phase was transferred to a new sample vial by a glass pipette. The residual n-hexane in sample vials was dried completely by nitrogen at 40 °C, and chloroform was then added to 100 μL. The samples were vortexed for blending, and 1 ul homogenate was analyzed by gas chromatograph (agilent8890) equipped with a DB-23 column (30 m × 0.25 mm × 0.25 um; Agilent, Santa Clara, CA, USA) and a flame ionization detector. Helium (1.5 mL, 21 min) was used as a carrier gas. The initial temperature was set at 180 °C and kept for 1 min, increased at 3 °C/min to 240 °C, and held for 39 min. Qualitative analysis was based on 37 known fatty acid methyl ester mixed standards (NU-CHEK, Elysian, MN, USA).

### 4.7. Bimolecular Luminescence Complementation (BiLC) 

The generated constructs were transformed into *Agrobacterium tumefaciens GV3101* through the freeze–thaw method and the colonies growing on LB agar plates containing corresponding antibiotics were chosen for transient tobacco transformation. Transient tobacco transformation was performed as previously described [56]. Briefly, the overnight cultures were suspended in infiltration solution (Liquid Murashige and Skoog (MS) medium containing 10 mM MES, 10 mM MgCl_2,_ and 100 μM Acetosyringone, pH 5.6–5.7). The suspension solution was then infiltrated into tobacco leaves by a 1 ml disposal plastic syringe and the infiltrated plants were incubated for 48–72 h in the growth chamber. One millimolar of fluorescein potassium salt was sprayed on the abaxial surface of leaves before observation. Photographs were imaged with the Tanon 5200 luminescence imaging system.

### 4.8. Water Loss Rate Assay

Rosette leaves of 4-week-old plants were placed immediately in water (in the dark) and soaked for 60 min. Leaves or stems were removed from soaking, shaken gently, and blotted to remove excess water, and weights were determined gravimetrically every 20 min using a microbalance in complete darkness. Water loss rates were recorded over 120 min and measured as a percentage of the initial weight of fully hydrated rosettes.

## 5. Conclusions

Using the CRISPER/Cas9 method, we created *kcs5* and *kcs6* single as well as double mutants and identified their roles in wax synthesis. The wax phenotype analysis of *kcs5* mutants revealed decreases in the total amounts of wax components derived from carbon 32 (C32) and C34 in leaves and C26 to C32 derivatives in flowers. Moreover, the simultaneous repression of both gene activity had additive effects on the wax biosynthesis process, suggesting the redundant roles of *KCS5* and *KCS6* during this process. Additionally, concurrent mutations of both *KCS5* and *KCS6* genes nearly block drought-induced wax production, implicating their key roles in wax biosynthesis during drought.

## Data Availability

Data are available from the authors on request.

## Supplementary Materials

The following supporting information can be downloaded at: https://www.mdpi.com/article/10.3390/ijms23084450/s1.
