# Supplementary material for: ArabidopsisKCS5 and KCS6 Play Redundant Roles in Wax Synthesis
Source: Int J Mol Sci. 2022 Apr 18;23(8):4450. doi: 10.3390/ijms23084450 (PMC9027390; doi:10.3390/ijms23084450)
Supplement: Supplementary file 1 [file ijms-23-04450-s001.zip › ijms-1657840-supplementary.pdf]

International Journal of *Molecular Sciences*

MDPI

Article

Arabidopsis KCS5 and *KCS6* Play Redundant Roles in Wax Synthesis

Haodong Huang [1,†], Asma Ayaz [1,†], Minglü Zheng [1], Xianpeng Yang [2], Wajid Zaman [3], Huayan Zhao [1,*] and Shiyou Lü [1,4,*]

[1] State Key Laboratory of Biocatalysis and Enzyme Engineering, School of Life Sciences, Hubei University, Wuhan 430062, China; haodonghuang@stu.hubu.edu.cn (H.H.); asmaayaz@bs.qau.edu.pk (A.A.); 202021107010975@stu.hubu.edu.cn (M.Z.)

[2] College of Life Sciences, Shandong Normal University, Jinan 250014, China; yangxp2006@sdnu.edu.cn

[3] Department of Life Sciences, Yeungnam University, Gyeongsan 38541, Korea. shangla123@gmail.com

[4] Hubei Hongshan Laboratory, Wuhan, 430070, China

[*] Correspondence: huayanzhao@hubu.edu.cn (H.Z.); shiyoulu@hubu.edu.cn (S.L.); Tel.:+86-27-88663882 (S.L)

[†] These authors contributed equally to this work.

Citation: Huang, H.; Ayaz, A.; Zheng, M.; Yang, X.; Zaman, W.; Zhao, H.; Lü, S. *Arabidopsis KCS5* and *KCS6* Play Redundant Roles in Wax Synthesis. *Int. J. Mol. Sci.* **2022**, *23*, 4450. https://doi.org/10.3390/ijms23084450

Academic Editor: Setsuko Komatsu

Received: 14 March 2022
Accepted: 14 April 2022
Published: 18 April 2022

Publisher's Note: MDPI stays neutral with regard to jurisdictional claims in published maps and institutional affiliations.

Copyright: © 2022 by the authors. Submitted for possible open access publication under the terms and conditions of the Creative Commons Attribution (CC BY) license (https://creativecommons.org/licenses/by/4.0/).

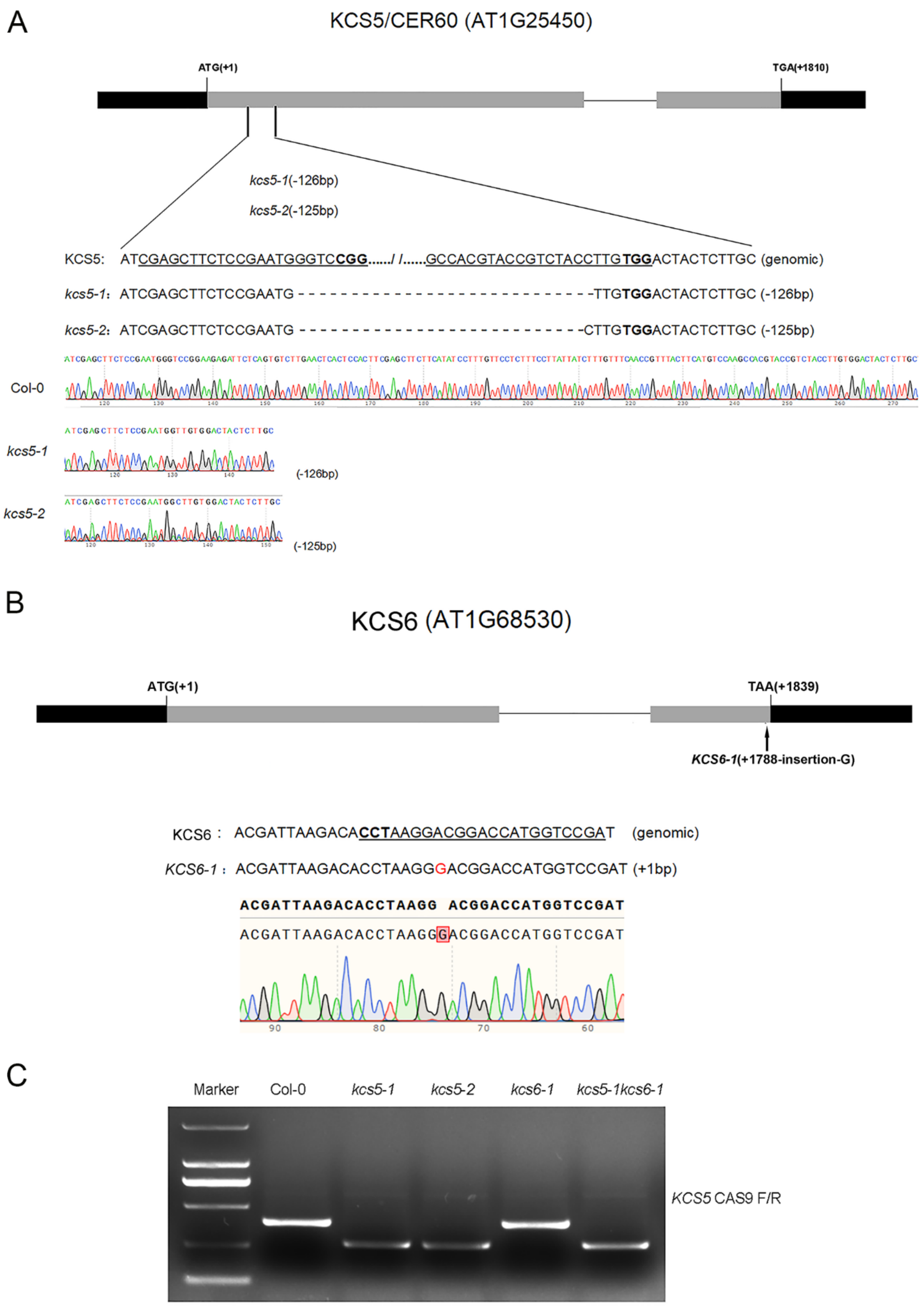

Figure S1. Molecular identification of *kcs5-1*, *kcs5-2*, *kcs6-1* and *kcs5-1 kcs6-1*. (**A**,**B**), *KCS5* and *KCS6* were mutated by CRISPR-Cas9 technology, and the mutation sites were confirmed by sequence. (**C**) The mutations in *kcs5-1*, *kcs5-2*, *kcs6-1* and *kcs5-1 kcs6-1* were identified by PCR.

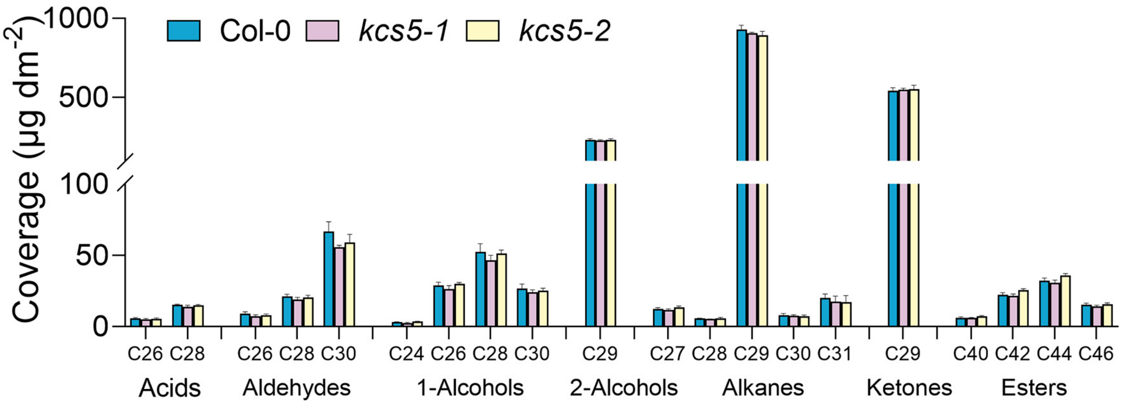

Figure S2. Stem wax profiles of Col-0, ***kcs5-1* and *kcs5-2*.** Wax coverage was expressed as wax amounts per stem surface area (μg × dm^{-2}). Each wax constituent was designated by carbon chain length and was labelled by chemical class along the x axis. Data are means ± SD (*n* = 5).

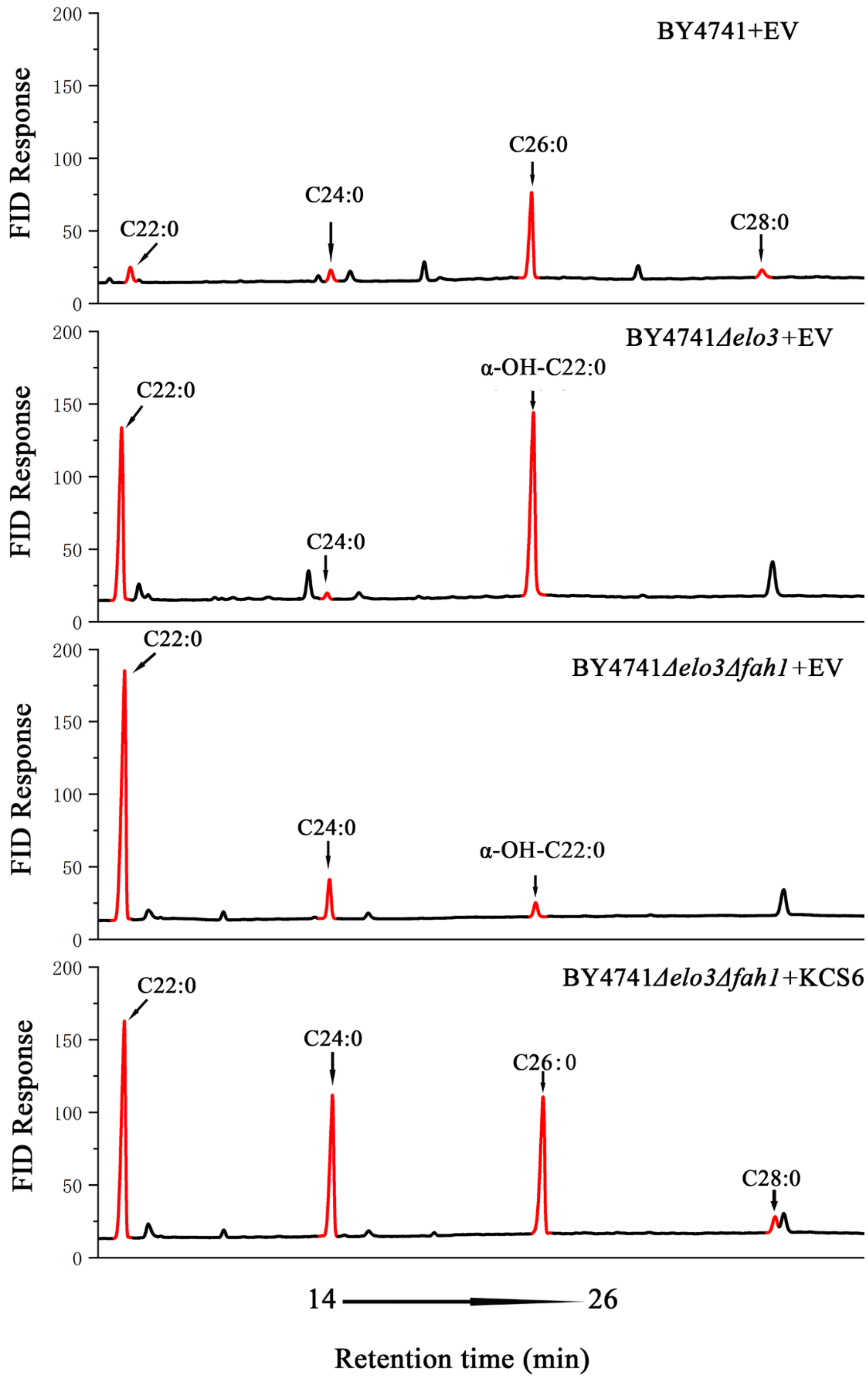

Figure S3. Gas chromatograms of yeast VLCFAs. Fatty acids were extracted from BY4741 transformed with empty vector (EV), BY4741*Δelo3* transformed with EV, BY4741*Δelo3Δfah1* transformed with EV or *KCS6*, converted to methyl esters, and analyzed by GC-MS. VLCFAs and *α*-OH-C22:0 was shown in red peaks.

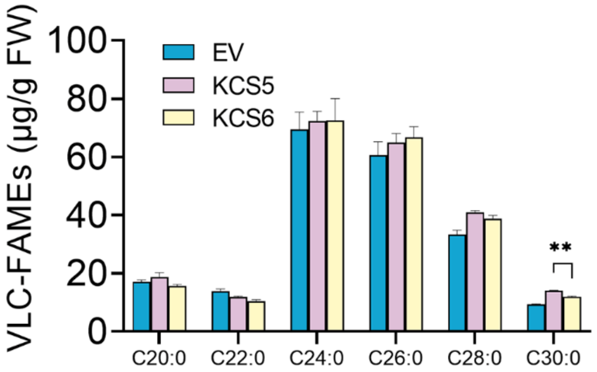

Figure S4. Fatty acid analysis in tobacco leaves transformed with Empty vector (EV), *KCS5* and *KCS6*. *EV*, *KCS5* and *KCS6* were transiently infiltrated into tobacco leaves. Fatty acid analysis was performed five days after infiltration. The values shown are mean±SD ($n = 4$). $^{**}p < 0.01$.

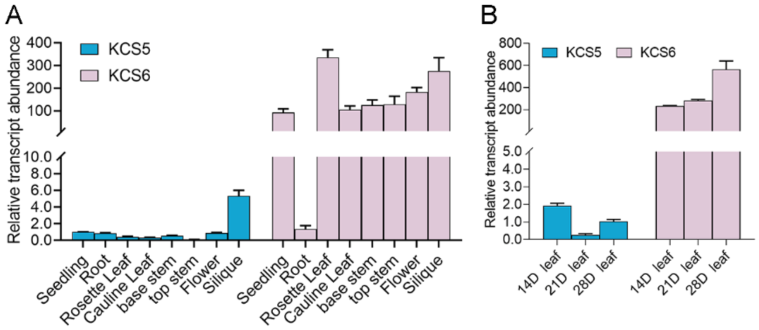

Figure S5. Expression patterns of *KCS5* and *KCS6* in different organs and in response to water deficits.

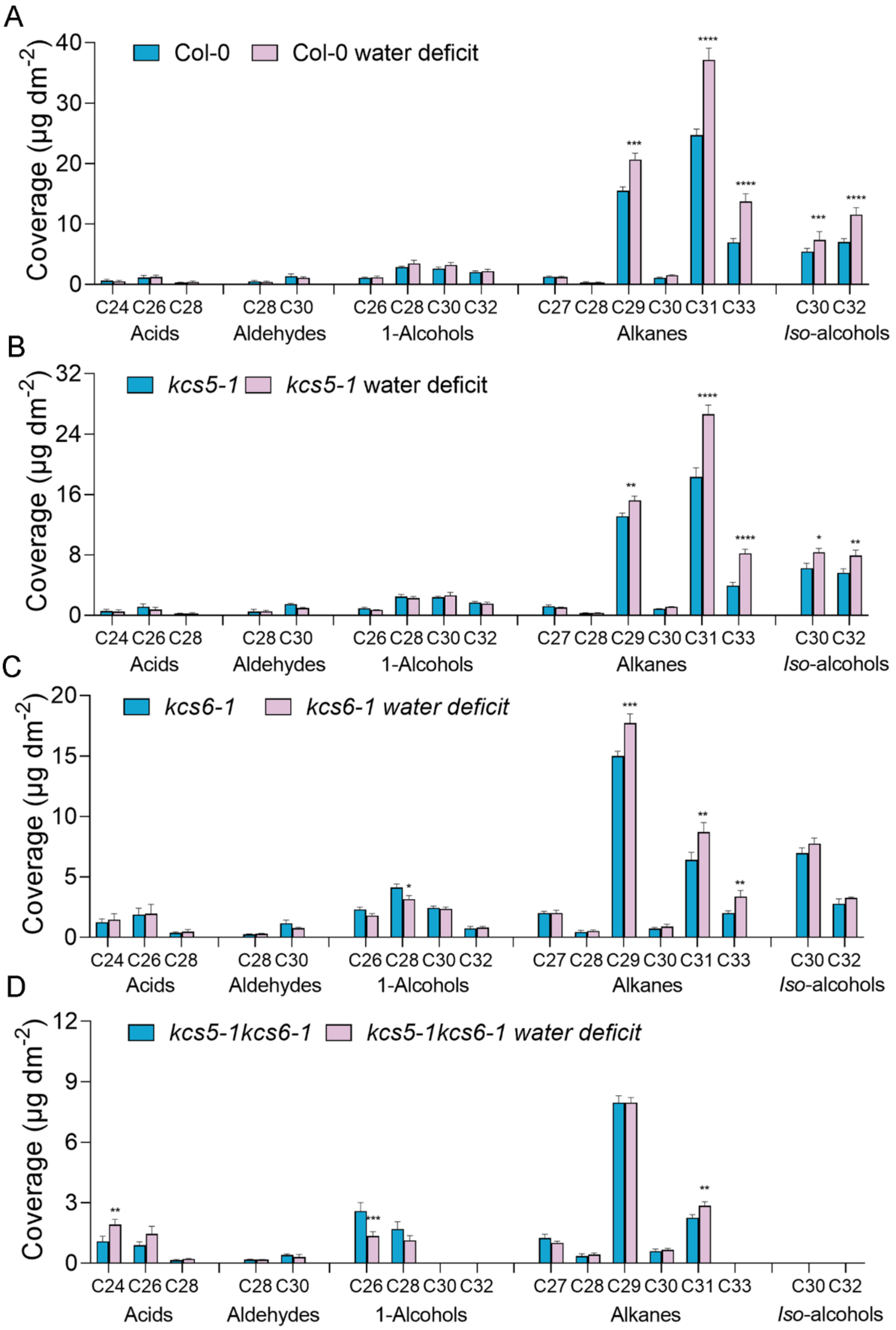

Figure S6. Wax profiles of Col-0, *kcs5-1*, *kcs6-1* and *kcs5-1 kcs6-1* rosette leaves under normal and water-deficit conditions. Wax coverage was expressed as wax amounts per leaf surface area (μg × dm^{-2}). Each wax constituent was designated by carbon chain length and was labelled by chemical class along the x axis. The values shown are mean ± SD ($n = 5$). * $p < 0.05$; ** $p < 0.01$; *** $p < 0.001$.

Table S1. Cuticular wax composition of rosette leaves, stems and flowers of *Arabidopsis* Col-0, *kcs5-1*, *kcs5-2*, *kcs 6-1* and *kcs5-1 kcs6-1* under normal condition or drought. Values shown are means ± SD ($\mu g/dm^2$) or (μg/mg FW), total wax amounts, and coverage of individual compound classes (n = 5). -, Undetectable. The values shown are mean ± standard deviation (n = 5).

Sample	Total	Fatty Acids	Aldehydes	1-Alcohols	2-Alcohol	Alkanes	Ketone	Esters	*iso*-Alcohols	*iso*-Alkanes
**Rosette leaves (μg/dm2)**										
**Col-0**	74.99 ±1.70	2.07 ±0.65	1.85 ±0.48	8.65 ±0.43	-	49.98 ±0.96	-	-	12.44 ±1.09	-
kcs5-1	61.02 ±1.23	1.95 ±0.69	1.99 ±0.37	7.50 ±0.52	-	37.70 ±2.04	-	-	11.89 ±1.14	-
kcs5-2	62.22 ±1.32	2.78 ±0.85	2.38 ±0.28	7.66 ±0.50	-	38.76 ±0.87	-	-	10.64 ±1.26	-
kcs6-1	51.26 ±2.76	3.53 ±0.79	1.43 ±0.25	9.61 ±0.59	-	26.58 ±0.83	-	-	10.10 ±0.99	-
kcs5-1 kcs6-1	19.45 ±0.69	2.13 ±0.44	0.59 ±0.07	4.29 ±0.78	-	12.44 ±0.35	-	-	-	-
**Leaves after drought treatment ($\mu g/dm^2$)**										
**Col-0**	107.46 ±1.86	2.18 ±0.60	1.53 ±0.04	10.18 ±1.32	-	74.66 ±3.59	-	-	18.91 ±2.51	-
kcs5-1	78.98 ±2.13	1.55 ±0.62	1.48 ±0.27	7.16 ±0.85	-	52.53 ±2.19	-	-	16.26 ±1.22	-
kcs6-1	56.98 ±0.94	3.90 ±1.41	1.05 ±0.10	8.13 ±0.53	-	33.26 ±1.96	-	-	10.64 ±1.04	-
kcs5-1 kcs6-1	19.51 ±0.45	3.55 ±0.69	0.48 ±0.14	2.49 ±0.37	-	12.94 ±0.50	-	-	-	-
**Stems ($\mu g/dm^2$)**										
**Col-0**	2057.91 ±54.54	21.44 ±0.48	97.02 ±7.63	111.56 ±11.38	230.70 ±8.10	976.73 ±28.70	543.91 ±18.06	76.54 ±4.97	-	-
kcs5-1	2000.92 ±25.30	18.96 ±2.00	82.11 ±2.94	100.16 ±7.72	228.87 ±3.74	949.37 ±3.85	548.25 ±9.63	73.22 ±4.02	-	-
kcs5-2	2028.35 ±66.77	20.61 ±0.93	87.95 ±6.87	110.70 ±4.85	233.11 ±6.51	937.25 ±30.44	553.84 ±22.94	84.89 ±3.58	-	-
kcs6-1	274.91 ±2.50	25.96 ±0.97	32.83 ±4.35	95.57 ±7.01	0.60 ±0.12	12.71 ±2.07	2.70 ±0.29	104.55 ±7.50	-	-
kcs5-1 kcs6-1	204.68 ±8.45	17.44 ±1.08	23.05 ±2.46	72.65 ±5.05	0.43 ±0.05	2.91 ±0.26	1.13 ±0.17	87.07 ±3.05	-	-
**Flowers (μg/mg FW)**										
**Col-0**	633.49 ±26.85	2.99 ±0.17	16.36 ±0.86	30.16 ±2.21	94.98 ±4.42	361.01 ±14.57	119.77 ±5.81	-	-	8.20 ±0.44
kcs5-1	534.38 ±13.45	2.52 ±0.18	14.05 ±0.96	25.69 ±1.03	77.84 ±1.04	298.89 ±9.28	110.71 ±2.97	-	-	4.67 ±0.66
kcs5-2	515.79 ±10.21	2.65 ±0.20	14.36 ±0.54	24.69 ±0.35	75.17 ±1.89	285.73 ±6.99	108.28 ±2.59	-	-	4.91 ±0.31
kcs6-1	90.49 ±1.49	2.90 ±0.45	7.30 ±0.55	13.22 ±0.94	5.28 ±0.30	56.35 ±1.57	3.91 ±0.30	-	-	1.52 ±0.08
kcs5-1 kcs6-1	33.16 ±2.37	2.27 ±0.36	5.06 ±1.36	8.20 ±0.34	2.75 ±0.09	11.78 ±0.36	2.68 ±0.04	-	-	0.43 ±0.06

Table S2. Fatty acids analysis of KCS5, KCS6, KCS5 and CER2, KCS5 and CER26, KCS6 and CER2, KCS6 and CER26 heterologously expressed in yeast (μg/OD600) and tobacco (μg/mg FW). The values shown are mean ± standard deviation (*n* = 5). -, Undetectable.

Sample	C20:0	C22:0	C24:0	C26:0	C28:0	C30:0	C32:0	C34:0
				**Yeast**				
**EV**	1.026 ± 0.07	6.234 ±0.62	0.800 ±0.05	0.321 ±0.01	-	-	-	-
KCS5	1.058 ±0.06	3.033 ±0.28	1.411 ±0.07	2.322 ±0.25	0.431 ±0.07	0.334 ±0.01	-	-
KCS6	0.901 ±0.07	3.448 ±0.35	2.210 ±0.17	2.319 ±0.25	0.289 ±0.05	0.160 ±0.01	-	-
KCS5 CER2	0.866 ±0.03	2.826 ±0.15	1.200 ±0.02	2.380 ±0.13	0.531 ±0.11	0.423 ±0.01	-	-
KCS6 CER2	0.763 ±0.06	3.034 ±0.26	1.716 ±0.09	1.511 ±0.09	0.209 ±0.03	0.356 ±0.01	-	-
**KCS5 CER26**	0.698 ±0.10	2.002 ±0.23	0.911 ±0.12	1.786 ±0.25	0.207 ±0.03	0.195 ±0.03	0.248 ±0.06	0.131 ±0.03
**KCS6 CER26**	0.687 ±0.06	2.724 ±0.17	1.722 ±0.09	2.137 ±0.18	0.221 ±0.03	0.192 ±0.02	0.144 ±0.02	0.194 ±0.03
KCS5 CER2 CER26	1.031 ±0.10	3.148 ±0.41	1.202 ±0.08	2.209 ±0.24	0.347 ±0.06	0.588 ±0.03	0.380 ±0.08	0.258 ±0.04
KCS6 CER2 CER26	0.842 ±0.04	2.767 ±0.16	1.463 ±0.05	1.964 ±0.07	0.276 ±0.03	0.293 ±0.01	0.216 ±0.03	0.263 ±0.02
				**Tobacco**				
**EV**	17.06 ±0.70	13.92 ±0.74	69.37 ±6.02	60.59 ±4.69	33.35 ±1.46	9.35 ±0.20	-	-
KCS5	18.66 ±1.59	11.84 ±0.44	72.24 ±3.40	64.97 ±3.06	40.96 ±0.70	14.04 ±0.18	-	-
KCS6	15.60 ±0.63	10.41 ±0.59	72.55 ±7.54	66.79 ±3.59	38.74 ±1.18	11.97 ±0.29	-	-

Table S3. List of primers used in this study.

Name	Sequence	Purpose
GABI_804G08-LP	TAACGGTCGATACAATCGGAC	For genotyping *cer6-3* T-DNA insertion lines
GABI_804G08-RP	GTACTCGAGACTTCCCCGAAG	For genotyping *cer6-3* T-DNA insertion lines
GABI-pAC161-8409	ATATTGACCATCATACTCATTGC	T-DNA left-border primer
CER6-2-F	AGAATCCTTGAACGTTCTGGC	For genotyping *cer6-2* lines
CER6-2-R	GCTAGATCAACTGAGATCAGGC	For genotyping *cer6-2* lines
*CER6*CAS9-F	ATTACTATCAAGGCAACGAG	For genotyping *cer6-4* crispr-cas9 lines
*CER6*CAS9-R	CAAACCGTTACTAAACCGTT	For genotyping *cer6-4* crispr-cas9 lines
*KCS5*CAS9-F	ACACCTCATCATAGGCATAGC	For genotyping *kcs5-1*and*kcs5-2* crispr-cas9 lines
*KCS5*CAS9-R	TTCGACACTCTTGGGATTGTC	For genotyping *kcs5-1*and*kcs5-2* crispr-cas9 lines
CER6-CRISPR-F	ATATATGGTCTCGATTGGGCTCGCCAC-CGGTCAGACGTTTTAGAGCTAGAAATAGC	*CER6* Crispr-cas9 knockout vector construction
CER6-CRISPR-R	ATTATTGGTCTCTAAACAAGGACGGAC-CATGGTCCGCAATCTCTTAGTCGACTCTAC	*CER6* Crispr-cas9 knockout vector construction
KCS5-CRISPR-F	ATA-TATGGTCTCGATTGGAGCTTCTCCGAATGG GTCGTTTTAGAGCTAGAAATAGC	*KCS5* Crispr-cas9 knockout vector construction
KCS5-CRISPR-R	ATTATTGGTCTCTAAACCAAGGTAGACGG-TACGTGGCAATCTCTTAGTCGACTCTAC	*KCS5* Crispr-cas9 knockout vector construction

CER6 nLUC-F	acgggggacgagctcggtacATGCCTCAGGCAC-CGATG	*CER6* Dual luciferase complementation assay (BiLC) vector construction
CER6 nLUC-R	cgggacgcgtacgagatctggtcGAGTTT-GACAACTTCGGGAATAAAGACAG	*CER6* Dual luciferase complementation assay (BiLC) vector construction
KCS5 nLUC-F	atacgaacgaaagctctg-caggTCATAGTTTAACAACTTCAGGGA-TAAAGACAGG	*KCS5* Dual luciferase complementation assay (BiLC) vector construction
KCS5 nLUC-R	cgggacgcgtacgagatctggtcATGTCTGAT-TTCTCGAGCTCCG	*KCS5* Dual luciferase complementation assay (BiLC) vector construction
CER2-cLUC- F	acgcgtcccggggcggtacATGGAGGGAAGCCCAG-TGAC	*CER2* Dual luciferase complementation assay (BiLC) vector construction
CER2-cLUC -R	atacgaacgaaagctctgcaggTTATA-TAATCATATTAGTCACCTCCTCCTTGAG	*CER2* Dual luciferase complementation assay (BiLC vector construction
CER26-cLUC- F	acgcgtcccggggcggtacATGGGTCGATCTCAA-GAACAGGGAC	*CER26* Dual luciferase complementation assay (BiLC) vector construction
CER26-cLUC-R	atacgaacgaaagctctg-caggTCATGGCGCGATCAAACCAAACTTC	*CER26* Dual luciferase complementation assay (BiLC) vector construction
KCS5-P42X- F	cgacggattctagaactagtATGTCTGAT-TTCTCGAGCTCCG	*KCS5* Yeast heterologous expression vector con-struction
KCS5-P42X -R	aactaattacatgactcgag-TCATAGTTTAACAACTTCAGGGATAAAGA-CAGG	*KCS5* Yeast heterologous expression vector con-struction
CER6-P42X- F	cgacggattctagaactagtATGCCTCAGGCAC-CGATG	*CER6* Yeast heterologous expression vector con-struction
CER6-P42X -R	aactaattacatgactcgagTTAGAGTTT-GACAACTTCGGGAATAAAG	*CER6* Yeast heterologous expression vector con-struction
CER2-P42X- F	cgacggattctagaactagtATGGAGGGAAGCCCAG-TGAC	*CER2* Yeast heterologous expression vector con-struction
CER2-P42X -R	aactaattacatgactcgagTTATATAATCATATTAG-TCACCTCCTCCTTGAG	*CER2* Yeast heterologous expression vector con-struction
CER26-P42X- F	cgacggattctagaactagtATGGGTCGATCTCAA-GAACAGGGAC	*CER26* Yeast heterologous expression vector con-struction
CER26-P42X-R	aactaattacatgactcgagTCATGGCGCGATCAAAC-CAAACTTC	*CER26* Yeast heterologous expression vector con-struction
KCS5-qRT-F	AAGCGAGAGAAAGAGCGTTG	qRT-PCR, *KCS5* (AT1G25450)
KCS5-qRT-R	TGCCTATGATGAGGTGTGGA	qRT-PCR, *KCS5* (AT1G25450)
CER6-qRT-F	GTGAAGCCCTCAAGGCAAAC	qRT-PCR, *CER6* (AT1G68530)
CER6-qRT-R	CGAAGGCCAGCTTGAAATCC	qRT-PCR, *CER6* (AT1G68530)
KCS5 RT-F	TCTCCCATCTGCCAACTTTC	RT-PCR *KCS5* (AT1G25450)
KCS5 RT-R	TCGCTCGAGGATTCTCATTT	RT-PCR *KCS5* (AT1G25450)
ACTIN2-qRT-F	GCACCCTGTTCTTCTTACCGA	qRT-PCR (AT3G18780)
ACTIN2-qRT-F	CTTGGATGGCGACATACATAGC	qRT-PCR (AT3G18780)
ACTIN2-F	GTTGGTGATGAAGCACAATCCAAG	RT-PCR (AT3G18780)
ACTIN2-R	CTGGAACAAGACTTCTGGGCATCT	RT-PCR (AT3G18780)
